# Subject-specific computational modeling of DBS in the PPTg area

**DOI:** 10.3389/fncom.2015.00093

**Published:** 2015-07-14

**Authors:** Laura M. Zitella, Benjamin A. Teplitzky, Paul Yager, Heather M. Hudson, Katelynn Brintz, Yuval Duchin, Noam Harel, Jerrold L. Vitek, Kenneth B. Baker, Matthew D. Johnson

**Affiliations:** ^1^Department of Biomedical Engineering, University of MinnesotaMinneapolis, MN, USA; ^2^Department of Neurology, University of MinnesotaMinneapolis, MN, USA; ^3^Center for Magnetic Resonance Research, University of MinnesotaMinneapolis, MN, USA; ^4^Institute for Translational Neuroscience, University of MinnesotaMinneapolis, MN, USA

**Keywords:** pedunculopontine nucleus, deep brain stimulation, Parkinson's disease, non-human primate, finite element, diffusion tensor

## Abstract

Deep brain stimulation (DBS) in the pedunculopontine tegmental nucleus (PPTg) has been proposed to alleviate medically intractable gait difficulties associated with Parkinson's disease. Clinical trials have shown somewhat variable outcomes, stemming in part from surgical targeting variability, modulating fiber pathways implicated in side effects, and a general lack of mechanistic understanding of DBS in this brain region. Subject-specific computational models of DBS are a promising tool to investigate the underlying therapy and side effects. In this study, a parkinsonian rhesus macaque was implanted unilaterally with an 8-contact DBS lead in the PPTg region. Fiber tracts adjacent to PPTg, including the oculomotor nerve, central tegmental tract, and superior cerebellar peduncle, were reconstructed from a combination of pre-implant 7T MRI, post-implant CT, and post-mortem histology. These structures were populated with axon models and coupled with a finite element model simulating the voltage distribution in the surrounding neural tissue during stimulation. This study introduces two empirical approaches to evaluate model parameters. First, incremental monopolar cathodic stimulation (20 Hz, 90 μs pulse width) was evaluated for each electrode, during which a right eyelid flutter was observed at the proximal four contacts (−1.0 to −1.4 mA). These current amplitudes followed closely with model predicted activation of the oculomotor nerve when assuming an anisotropic conduction medium. Second, PET imaging was collected OFF-DBS and twice during DBS (two different contacts), which supported the model predicted activation of the central tegmental tract and superior cerebellar peduncle. Together, subject-specific models provide a framework to more precisely predict pathways modulated by DBS.

## Introduction

Gait and balance difficulties in Parkinson's disease can be especially debilitating since they increase the risk of falling. For some patients, these symptoms are resistant or poorly managed by levodopa treatment and typical targets of deep brain stimulation (DBS) including the subthalamic nucleus (STN) and internal segment of the globus pallidus. In contrast, low-frequency electrical stimulation delivered within or near the pedunculopontine tegmental nucleus (PPTg), a component of the mesencephalic locomotor region (MLR) of the brainstem, has shown promising results (Plaha and Gill, 2005; Schrader et al., 2013; Mazzone et al., 2014). Clinical outcomes, however, have varied from patient to patient across these studies, due in part to variation in surgical targeting, uncertainty in the therapeutic target, and the likely modulation of highly excitable, side effect inducing fiber pathways (Nowak and Bullier, 1998) outside the MLR.

A previous computational modeling study showed that clinical outcomes of DBS within the PPTg area are likely to be highly dependent upon lead position and stimulation settings (Zitella et al., 2013). For instance, the superior cerebellar peduncle passes through the PPTg en route from the deep cerebellar nuclei to the red nucleus and cerebellar receiving area of the motor thalamus by means of a decussation just rostral to PPTg. At present, how selective activation of this pathway affects freezing of gait is unknown, though stimulation of the cerebello-thalamo-cortical circuit has been postulated to be beneficial for gait (Fournier-Gosselin et al., 2013). DBS in the PPTg area may also modulate medial fiber tracts such as the medial longitudinal fasciculus (MLF) or the oculomotor nerve (ON). Side effects from activation of either of these fiber tracts would be expected to affect the eyes and eyelids. Neuronal activation volumes that extend lateral of PPTg may include the medial lemniscus (ML) and lateral lemniscus (LL) and lead to paresthesias (Murata et al., 2003) and changes in auditory perception (Lim et al., 2008), respectively. Further, spread of current rostral to the PPTg may modulate the central tegmental tract (CTG), which rises from the nucleus solitarius and carries gustatory input to the ventral posteromedial nucleus of the thalamus (VPM). There is some evidence that activation of this tract may result in palatal myoclonus (Matsuo and Ajax, 1979).

While previous subject-specific computational models of DBS have been developed for the STN (Miocinovic et al., 2006; Chaturvedi et al., 2010; Butson et al., 2011), tailoring models to the PPTg area has been limited because of the poor image contrast within brainstem with standard MR scanner technology. In recent years, however, advances in structural imaging have made visualizing fiber tracts within the brainstem more readily available. In this study, we leverage susceptibility-weighted imaging (SWI) and diffusion-weighted imaging (DWI) to create subject-specific computational models of PPTg-DBS, which can predict activation of individual fiber tracts within the brainstem for any given DBS setting. In order for these models to be informative for clinicians, the models must provide accurate predictions of neuronal activation. The challenge becomes defining behavioral or functional outcome measures to confirm or otherwise modify the selection of model parameters including tissue conductance anisotropy and inhomogeneity, cellular morphology, axonal diameter, and ion channel kinetics among others. Here, we propose two approaches in the context of PPTg-DBS, namely eliciting an oculomotor side effect and performing DBS within the context of positron emission tomography (PET) imaging.

To examine the pathways modulated in the PPTg area during PPTg-DBS, a subject-specific computational model was developed. In this study, the models were used to (1) investigate the effects of using tissue conductance anisotropy within the brainstem based on diffusion-weighted imaging, and (2) perform model parameter sweeps to determine PPTg-DBS model sensitivity. The models were evaluated with varying axon diameter, conductivity values, and DBS lead location, and then compared against behavioral and PET imaging results.

## Materials and methods

### Subject

Two female rhesus macaque monkeys (*macaca mulatta*, Monkey L and Monkey P) were used in this study. All procedures were approved by the Institutional Animal Care and Use Committee of the University of Minnesota and complied with United States Public Health Service policy on the humane care and use of laboratory animals. The animals were housed individually with environmental enrichment, provided with water *ad libitum*, and given a range of food options including fresh fruit and vegetables. All efforts were made to provide good care and alleviate any discomfort for the animals during the study.

Pre-operative 7T MRI was acquired at the Center for Magnetic Resonance Research (CMRR) at the University of Minnesota using a passively shielded 7T magnet (Magnex Scientific) for both animals. During the imaging sessions, the animals were anesthetized with isoflurane (2.5%) and monitored for depth of anesthesia. Susceptibility-weighted imaging was acquired with a 3D flow-compensated gradient echo sequence at 0.4 mm isotropic resolution using a field of view (FOV) of 128 × 96 × 48 mm^3^. Diffusion-weighted images (*b*-value = 1500 s/mm^2^) were acquired with diffusion gradients applied along 110 uniformly distributed directions using a 128 × 84 × 99 mm^3^ FOV (1 mm isotropic resolution). The 3D tensors were calculated as ellipsoidal functions, to identify the orientation of maximum value (Barmpoutis et al., 2009; Barmpoutis and Vemuri, 2010).

In Monkey L, a cranial chamber was mounted on the head to facilitate implantation of the DBS lead, as described previously (Elder et al., 2005). The high-field imaging, along with results from electrophysiological microelectrode mapping of the PPTg area, were superimposed in Monkey Cicerone (Miocinovic et al., 2007) to define a trajectory for unilateral implantation of a scaled-down version of a human DBS lead (2F diameter, 8 annular electrode contacts: 0.5 mm height, 0.25 mm spacing) (NuMed, Hopkinton, NY) in the region of the PPTg (right hemisphere). Following lead implantation, a post-operative CT scan was performed under Ketamine and Dexdomitor anesthesia to visualize the implantation trajectory and depth in Monkey Cicerone. The preoperative SWI was co-registered with the postoperative CT to determine the DBS lead location relative to nuclei and fiber tracts within the brainstem. After instrumentation with the chamber and the DBS lead, the subject was rendered parkinsonian with systemic injections of 1-methyl-4-phenyl-1,2,3,6-tetrahydropyridine (MPTP).

At the conclusion of the study, both animals were deeply anesthetized with sodium pentobarbital and perfused with a fixative solution containing 4% paraformaldehyde, consistent with the recommendations of the Panel of Euthanasia of the American Veterinary Medical Association. After fixation, the brain was removed, blocked, and cryoprotected in 15% sucrose in phosphate buffered solution. Coronal sections (50 μm) were cut using a freezing microtome and labeled for Nissl. In the case of Monkey L, the DBS lead trajectory was again reconstructed from these histological images using Mimics (Materialise, Leuven, Belgium), which confirmed the *in vivo* estimation of the DBS lead trajectory that had been generated from co-registration of the pre-implant SWI with the post-implant CT.

### Axonal model morphologies

**Figure 1:**
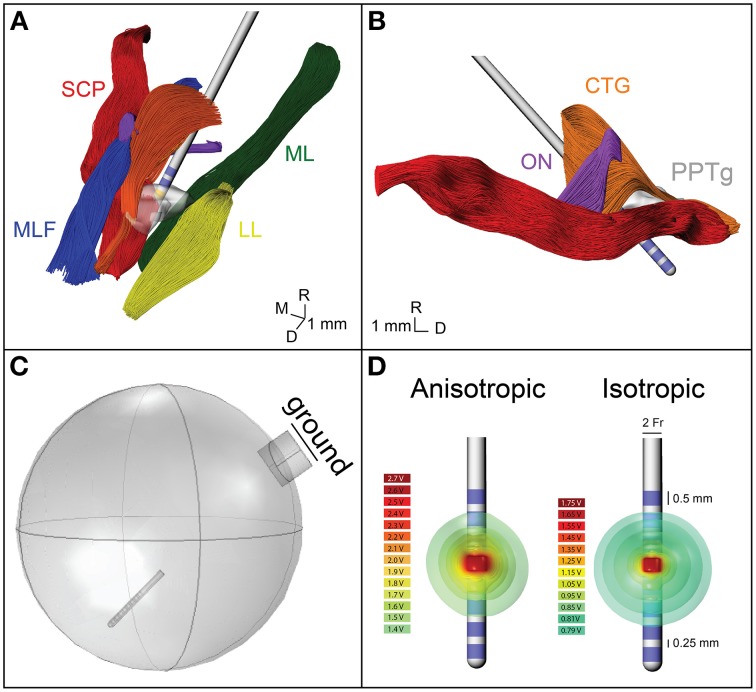
**Model geometry and FEM. (A)** The geometry of the fiber pathways in the PPTg area in relation to the DBS lead location. CTG, central tegmental tract—orange; ON, oculomotor nerve—purple; SCP, superior cerebellar peduncle—red; MLF, medial longitudinal fasciculus—blue; ML, medial lemniscus—green; LL, lateral lemniscus—yellow; PPTg, pedunculopontine tegmental nucleus—gray. **(B)** Sagittal view of the geometry of the modeled fiber pathways. **(C)** The FEM geometry, showing the lead location and grounded chamber. **(D)** Electric potential isosurfaces for the anisotropic and isotropic model.

Several imaging-based tools were used to reconstruct the three-dimensional morphologies of the PPTg area for use in the subject-specific finite element model and multi-compartment neuron model simulations (Figures 1A,B). The SWI volume was aligned in anterior commissure to posterior commissure (AC-PC) space with Analyze (AnalyzeDirect, Overland Park, KS), and then resliced to generate images that matched atlas plates from a rhesus macaque brain atlas (Paxinos, 2009). A nonlinear affine atlas registration algorithm based on a moving least squares fit applied to each image voxel was used to identify the borders of the PPTg, CTG, and ON. The algorithm involved the selection of analogous control points placed on each MR image and each corresponding atlas plate. Contours were traced from these warped atlas reconstructions in Rhinoceros (McNeel, Seattle, WA) and lofted into 3D surfaces using a non-uniform rational B-spline modeling approach. Results from the warping algorithm were aligned to the DWI using FLIRT, which provided anatomical context to guide the placement of seed points for probabilistic diffusion tractography calculations in FSL (Jenkinson et al., 2012). To identify the SCP tract, seed points were placed in the cerebellar outflow tract caudal to the decussation, with waypoints defined at the decussation of SCP and the entire contralateral thalamus. Manual thresholding of the output of probtrackx was performed in Amira (FEI, Hillsboro, OR) to produce the final tract geometry.

### Finite element models (FEM)

A finite element model of the DBS lead and surrounding neural tissue was created in COMSOL (Figure 1C). The variable resolution tetrahedral mesh was constructed with a maximum element size of 0.2 mm for the electrodes and 1.6 mm for the lead, encapsulation layer, and neural tissue. The final mesh consisted of 447280 elements with a finer resolution near the electrode-tissue interface. A point current source was modeled at the geometric center of each electrode, and the entire lead was surrounded by a 250 μm encapsulation layer with a conductivity of 0.18 S/m. A 20 mm radius sphere around the electrode represented the neural tissue. A cylinder was placed on the edge of the sphere to represent the cranial chamber, and the chamber outer surface was assigned as ground (Figure 1C). For the isotropic FEM, conductivity of the neural tissue was homogeneous, 0.3 S/m. For the anisotropic FEM, the neural tissue conductivity was calculated from the 6-direction DTI tensors, based on an estimated linear relationship between the conductivity (σ) and diffusion tensor eigenvalues (Tuch et al., 2001).

**Figure 2:**
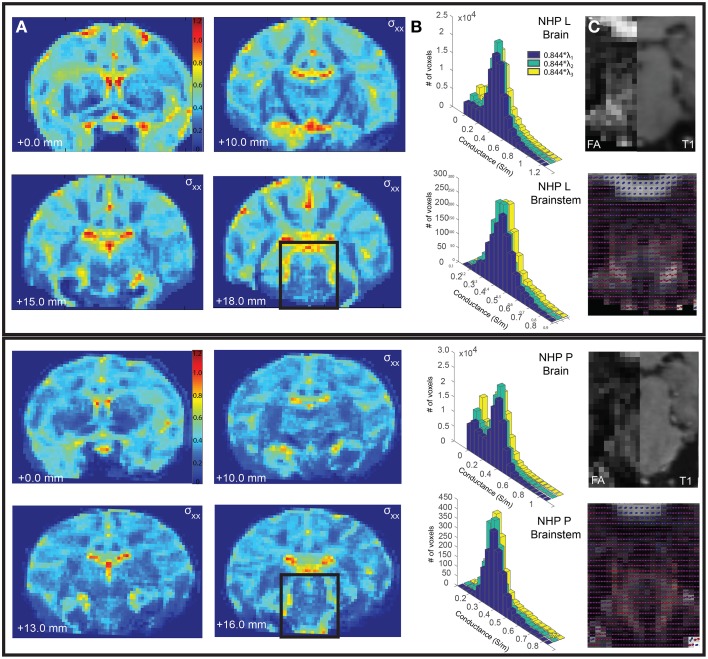
**Comparison of conductivity and diffusion tensors between Monkey L (top) and Monkey P (bottom). (A)** The calculated conductivity, σ_xx_, is shown for select coronal slices. **(B)** The distribution of conductivity values calculated from the primary, secondary, and tertiary eigenvalues for the entire brain (top) and the segmented brainstem (bottom). **(C)** The fractional anisotropy for a select brainstem slice (left), compared to a corresponding T1 slice (right). The diffusion tensors are plotted as spherical functions and overlaid on the fractional anisotropy. The orientations (dorsal-caudal, anterior-posterior, medial-lateral) are represented as RGB color components (i.e., R, G, and B, respectively).

(1) σ=s*D

where s was set to 0.844 and D represents the diffusion tensor eigenvalues. These conductivity matrices (Figures 2A,B) were imported into COMSOL and interpolated onto the mesh using the nearest neighbors function. Voltage distribution during monopolar stimulation through each electrode were solved for using anisotropic and isotropic conductivity models with COMSOL (Figure 1D) (Schmidt and Van Rienen, 2012). The extracellular voltage predicted from the FEM solution was then interpolated at each nodal compartment along each multi-compartment axon model.

### Biophysical modeling of DBS in the PPTg area

SCP, ON, and CTG were each randomly populated with multi-compartment cable models of 1000 myelinated axons, ranging in diameter from 2 to 8.7 μm (McIntyre et al., 2004a; Miocinovic et al., 2006; Johnson and McIntyre, 2008; Birdno et al., 2011). The axonal models included nodes of Ranvier, paranodal, and intermodal segments as well as a myelin sheath (McIntyre et al., 2002). Nodal compartments were given biophysical mechanisms related to a nonlinear fast Na^+^ channel, persistent sodium channel, slow K^+^ channel, and a leakage current. The paranodal compartments were instantiated with a slow K^+^ current. Both intermodal and myelin compartments had only passive mechanisms, including a membrane capacitance (Cm = 2 μF/cm^2^) and axoplasmic resistivity (70 Ω-cm). Stimulus thresholds for evoking action potentials within the modeled axons were estimated in the NEURON v7.3 programming environment (Hines and Carnevale, 1997). Stimulus perturbations were inserted using the NEURON mechanism, *extracellular*, for each axonal compartment. Stimulus pulses (90 μs pulse width) were delivered at a rate of 20 Hz. Activation was defined by the lowest amplitude to elicit one or more action potentials within 1–3 ms following each stimulus pulse for at least 8 of 10 stimulus pulses.

### Motor side-effects of DBS in the PPTg area

A monopolar review was conducted to determine the electrical stimulation amplitude thresholds for eliciting an overt motor side effect, which for this lead implantation was found to be a right eyelid flutter. In this case, stimulation was delivered through an externalized current-driven pulse generator (Precision, Boston Scientific) with a cathode applied individually to each contact and a return set to the cranial chamber. The stimuli were delivered as a 20 Hz train of 90 μs pulses in 0.1 mA increments until the eyelid flutter was observed visually by the investigators, as shown in Table 1.

**Table 1:** **Motor side-effect thresholds**.

**Contact**	**Stimulation frequency (Hz)**	**Threshold (mA)**	**Side effect**
7	20	1.0	R eyelid flutter
6	20	1.1	R eyelid flutter
5	20	1.4	R eyelid flutter
4	20	1.4	R eyelid flutter

### PET analysis

Approximately 1 year after implantation of the DBS lead in Monkey L, PET/CT was collected using a Siemens Biograph 64 slice scanner on three different days within an 8-day period. A full 24 h before each scan, the subject was withheld from any stimulation or medication. The subject was fasted beginning at 1700 the night prior to the scan (Garraux et al., 2011), with fasting blood glucose verified the morning of the scan. Thereafter, the proximal end of the DBS lead was connected to the external pulse generator and a single 8 mCi dose of 18-FDG was administered intramuscularly. As listed in Table 2, immediately following this injection either 20 Hz PPTg-DBS was applied at one of the two contacts of the DBS lead or no DBS was applied (baseline scan). The subject sat still in a quiet, familiar environment without stimuli while this treatment was administered. After 45 min, general anesthesia was induced using ketamine (10 mg/kg) and diazepam (0.5 mg/kg) (Oguchi et al., 1982; Wyckhuys et al., 2014), and the subject was moved to the scanner for imaging. Reconstruction yielded a voxel size of 1.018 × 1.018 × 2 mm. The subject was then released to an isolation room until radioactivity was undetectable.

**Table 2:** **DBS conditions during each PET scan**.

**Day**	**Condition**	**Parameters**
1: Day 0	Baseline, DBS OFF	No DBS
2: Day 2	Condition 1, DBS ON	Contact 7 at 0.9 mA, 20 Hz
3: Day 7	Condition 2, DBS ON	Contact 4 at 1.2 mA, 20 Hz

The PET images were analyzed with methods similar to those described previously (Ponto et al., 2014; Wyckhuys et al., 2014). Each non-contrast CT scan was transformed by rigid, manual co-registration (Jena et al., 2014) to align with a standard MRI template, INIA19 Macaca mulatta (Rohlfing et al., 2012), with the resultant transformation values individually applied to the PET image from the corresponding data acquisition session to align them to the normalized space. Finally, a preoperative MRI, taken before the cephalic hardware and DBS lead had been placed, was aligned with the INIA19 MRI to verify fit. The 1210-MRI-derived-VOIs of the INIA19 template, were used within PMOD software (PMOD Technologies, Zurich, Switzerland) to compile statistical measures. Mean voxel value, standard deviation, and number of voxels were collected as standard uptake values (SUV) (Garraux et al., 2011). All scans were scaled so that the left occipital white matter uptake was equivalent. Volumes of interest were then consolidated to yield larger brain structures, decreasing the resolution from 1210 volumes of interest into a manageable grouping for analysis. The scaled images had a two tailed *t*-test performed at each of the VOIs, with a non-corrected *p*-value (alpha = 0.05). All brain regions with a positive T score, corresponding to a relevant increase in brain activity, and *p* < 0.05 were analyzed further. These brain region SUVs are reported below along with respective T scores and *p*-values. Due to the use of a single subject, no CT transformation based image attenuation correction was performed on the PET scan results.

## Results

Accurate prediction of therapeutic outcomes by computational neuron models of DBS targeting the MLR of the brainstem will have strong clinical value for freezing of gait in Parkinson's disease patients with these implants (Zitella et al., 2013). Two major challenges remain in rendering these computational models more predictive in power: (1) making them subject specific, and (2) calibrating the model parameters. This work provides a framework to address both challenges using a combination of structural imaging at high magnetic fields, stimulus-evoked behavior using a monopolar review, and functional imaging.

### Conductivity anisotropy

A subject-specific model was created for Monkey L, but the conductivity, fractional anisotropy, diffusion tensors, and conductivity distributions were analyzed for both Monkeys L and P. Fractional anisotropy measured the difference between the three eigenvalues of the diffusion tensor (Pierpaoli et al., 1996). If the diffusion was isotropic (all three eigenvalues are equal), this value became 0. If a large number was calculated, there was high diffusion anisotropy. These values were scaled between 0 (isotropic) and 1 (anisotropic) and displayed as black and white, respectively (Figure 2C). In the brainstem region of Monkey L and Monkey P, the fractional anisotropy was found to be highly variable, with values ranging from less than 0.1–0.7. Since the voxel size of the DTI was 1 mm isotropic, each voxel could be composed of multiple fiber tracts, explaining this variability. The highest fractional anisotropy values (~0.5–0.7 in Monkey L, ~0.3–0.7 in Monkey P), appeared to correspond to areas of the superior cerebellar peduncle (caudal of the decussation) as well as the medial lemniscus, two of the largest pathways in the brainstem. However, the fractional anisotropy values for the selected slices in Figure 2C at the decussation of SCP were small (Monkey L: 0.295, 0.224, Monkey P: 0.181, 0.278, 0.268) and did not vary much from the mean of the surrounding voxels (Monkey L: 0.2867 ± 0.0752, Monkey P: 0.2575 ± 0.0504).

All six parameters of the diffusion tensor are visualized as spherical functions in Figure 2C. In both Monkey L and Monkey P, the greatest difference in the overall tensor direction in the brainstem was seen in the area of the ML, where the tensors were primarily dorsal-caudal and were oriented at a 45 degree angle from the surrounding voxels. Additionally, the principal direction (V1) at the decussation of the SCP was oriented medial-lateral, with a 90° difference compared to the neighboring voxels. When comparing neighboring voxels elsewhere in the brainstem, many midline voxels displayed at least a 45° difference in the longest axis. While some variability between animals was expected, the overall anisotropy in the brainstem was comparable.

Given that the brainstem was composed of a heterogeneous set of nuclei and fiber tracts, we hypothesized that an FEM of the brainstem with anisotropic conductivity would exhibit strong asymmetries in comparison to an otherwise equivalent isotropic model. The conductivity values, σ_xx_, derived from the subject-specific imaging are shown in Figure 2A for several coronal sections throughout the brain. Histograms of the conductivity values calculated from the primary, secondary and tertiary eigenvalues were given for the entire brain, including ventricles, and for the brainstem region around the DBS lead (Figure 2B). This region included the pons and part of the midbrain, demarcated as posterior of the substantia nigra. The average calculated conductivity along the main axes in the brainstem was between 0.3235 and 0.4018, just above 0.3 S/m, the value used for the isotropic models.

When conductivity anisotropy was incorporated into the models, the spread of current in the tissue was altered. As seen in Figure 1D, the isosurfaces of the electric potential in the isotropic model were spherical, while the isosurfaces in the anisotropic model were non-spherical. This is consistent with previous modeling studies that incorporated anisotropy (Miocinovic et al., 2009). Model predictions for activation threshold were much lower for anisotropic models than for the isotropic model. For example, the threshold for activating 5% of CTG axons using contact 7 was 0.5 mA for the anisotropic model and 1.1 mA for the isotropic model.

**Figure 3:**
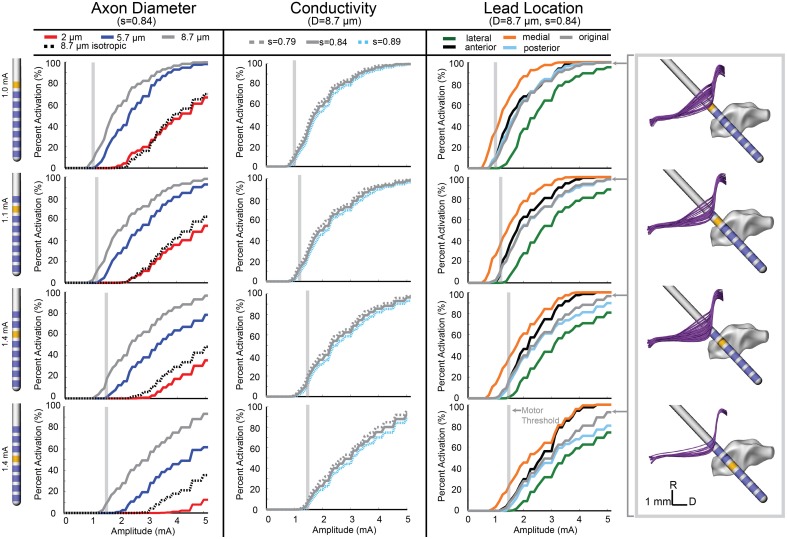
**Model-predicted activation of the ON fiber tract**. Percent activation is plotted for each stimulation amplitude. Each column shows the variability of model predictions when changing axon diameter, conductivity scaling factor (s), and lead location. (Right) The axons activated at the motor threshold current for each contact are plotted.

**Figure 4:**
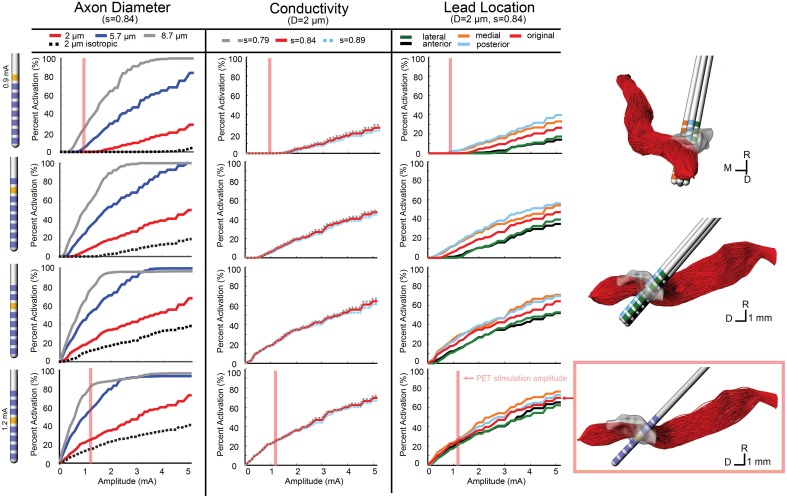
**Model-predicted activation of the SCP fiber tract**. Percent activation is plotted for each stimulation amplitude. Each column shows the variability of model predictions when changing axon diameter, conductivity scaling factor (s), and lead location. (Right) The 0.5 mm lead displacement is shown in the context of the SCP axons. The axons activated at the PET stimulation amplitude for contact 4 (configuration 2) is shown.

**Figure 5:**
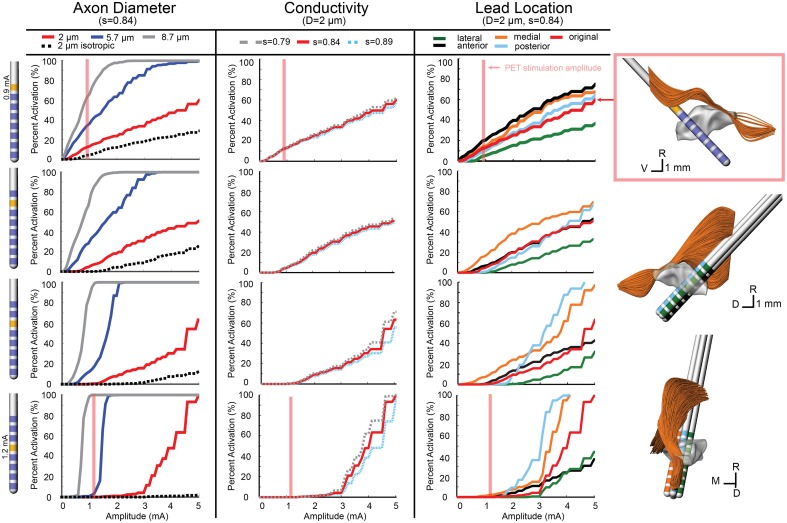
**Model-predicted activation of the CTG fiber tract**. Percent activation is plotted for each stimulation amplitude. Each column shows the variability of model predictions when changing axon diameter, conductivity scaling factor (s), and lead location. (Right) The 0.5 mm lead displacement is shown in the context of the CTG axons. The axons activated at the PET stimulation amplitude for contact 7 (configuration 1) is shown.

The conductivity scaling factor (s) has been previously reported as the range of *s* = 0.844 ± 0.0545 S·s/mm^3^ (Tuch et al., 2001) with varying scaling factors used in other modeling studies (McIntyre et al., 2004b; Butson et al., 2006). To investigate model sensitivity to the conductivity scaling factor within the reported range, activation threshold curves were generated using three values for s (0.79, 0.844, and 0.89). These results are shown in the second column for the ON (Figure 3), SCP (Figure 4), and CTG (Figure 5) tracts assuming an 8 μm diameter axonal fiber for ON and a 2 μm diameter for CTG and SCP. Varying the scaling factor ±6.5% resulted in only a minor shift (0.0981 mA) in the threshold for 5% activation of ON fibers using contact 7.

### Model parameter sweep

In addition to investigating model sensitivity to tissue conductivity, other model parameters known to impact the calculation of activation thresholds such as axon diameter (Rattay, 1999) and precise lead location were investigated as well. Previous models of axons in the brainstem region (SCP, medial lemniscus, lateral lemniscus) were modeled with a diameter of 2 μm (Zitella et al., 2013). While this is a conservative estimation for SCP and CTG axonal diameters, we also examined the effects on activation thresholds when using 5.7 and 8.7 μm axon diameter, which may be more representative of actual axon diameters within the SCP (Hazrati and Parent, 1992) and CTG tracts. The ON tract was also modeled with 2, 5.7, and 8.7 μm axon diameter with the latter thought to be the most realistic axon diameter. Atomic force microscopy has shown human oculomotor nerve fibers with much larger diameter fibers, between 10 and 15 μm (Melling et al., 2003), which presumably would be slightly smaller in the rhesus macaque. Consistent with the principles of cable models of myelinated axons, the axonal diameter had a large effect on the resultant activation thresholds for all three fiber tracts (Figures 3–5).

Model sensitivity to the precise position of the DBS lead within the brainstem was investigated by shifting the lead in four directions. Using the same implantation angle as defined by the SWI/CT co-registration process and histological reconstructions from Monkey L, the DBS lead was shifted 0.5 mm anterior, poster, medial, and lateral of the original lead placement (chamber reference). Moving the lead medially increased ON activation, while moving the lead laterally decreased ON activation. At the threshold for contact 7 (1 mA), the model predicted a 27.5% increase in ON activation for a medial lead location. Overall, anterior and posterior deviation in lead location did not alter the model results for amplitudes near the threshold. Predicted activation from stimulation through contact 5 at 1.4 mA only decreased by 0.7% when the lead was moved in the anterior direction (Figure 3).

For contacts 5, 6, and 7, SCP activation increased when the lead position was shifted medial and posterior, while an anterior and lateral lead position decreased activation. SCP activation at 0.9 mA through contact 7 increased the activation by 2.1% (from 0 to 2.1%) when moving the lead posterior. Contact 4 was embedded within SCP, so lead location had minimal effect on SCP activation at lower amplitudes (below 2 mA). However, for amplitudes above 2 mA, the anterior and lateral lead placements decreased activation and the medial lead placement increased activation. For 0.9 mA stimulation through contact 4, change in SCP activation was negligible (~0.1%) (Figure 4).

Due to the anatomy of the CTG, the effect of lead location was different for each active contact. In the posterior direction, there was minimal change in activation for contacts 6 and 7. The same was true for contact 4, at stimulation amplitudes below 1.6 mA. Contact 5 stimulation produced lower activation with a posterior lead placement until stimulation amplitude increased beyond 2 mA, which resulted in a large increase in activation that exceeded the original lead placement results. For all contacts, lateral shift decreased activation and medial shift increased activation, but the magnitude of these changes in activation differed for each contact. For the CTG, moving the lead 0.5 mm medially increased tract activation by 8% when stimulating through Contact 7 at 0.9 mA and 2.6% when stimulating through Contact 4 (Figure 5).

### Comparison of ON model simulations to stimulus-induced eyelid flutter

A monopolar review was conducted across all 8 contacts by applying a 20 Hz train of 90 μs pulses in increasing amplitude at intervals of 0.1 mA until a motor side effect was observed or the amplitude of stimulation reached 3.5 mA. For the proximal four electrodes (contacts 4–7), a right eyelid flutter was observed at amplitudes at or above 1 mA (Table 1) with more proximal contacts requiring higher stimulation amplitudes. At 20 Hz stimulation, the therapeutic PPTg-DBS stimulation frequency, stimulation resulted in an eyelid flutter, while stimulation at higher frequencies (e.g., 130 Hz) resulted in the eyelid remaining elevated. No other overt motor signs were observed at any of the stimulation amplitudes tested for other contacts.

The oculomotor nerve is known to project to the levator palpabrae superioris muscle of the eye, which is responsible for elevation of the upper eyelid (Porter et al., 1989). Multi-compartment axon models were developed for the oculomotor nerve to identify model parameter settings that resulted in the most consistent activation values across the empirical motor threshold amplitude values as defined in Table 1. We assumed that the neuron models should predict an activation of 5–15% based on previous models of the corticospinal tract of internal capsule (Chaturvedi et al., 2010) at the experimental motor threshold. Using the ON computational model (diameter = 8.7 μm, *s* = 0.844, original lead location), the percentage of activated axons at motor thresholds was calculated (Table 3). For each behavioral threshold, the percent error was calculated as the difference between the experimental threshold and the model-predicted stimulation amplitude necessary to activate 5% of the axons. There was no error in the model predictions for contact 5, 6, and 7. The percent error for contact 4 was 6.67%.

**Table 3:** **Comparison of ON model simulations to behavior thresholds**.

**Behavioral thresholds**	**% Activated at motor threshold**	**% Error**
C7 (1.0 mA)	7.7	0
C6 (1.1 mA)	8.7	0
C5 (1.4 mA)	9.7	0
C4 (1.4 mA)	2.9	6.67

The neuron modeling results from the anisotropic model resulted in much lower activation thresholds than were predicted from the isotropic model. Moreover, the anisotropic models, in comparison to the isotropic models, resulted in activation thresholds that were more consistent with the thresholds for inducing eyelid flutter (Figure 3). For the isotropic models, there was 0% activation of ON at the threshold amplitude for all contacts. Using contact 5, a 5% activation of ON fibers was achieved at 1.25 mA for the anisotropic model, while the isotropic model required 3.4 mA to reach 5% activation. Similarly for contact 6, 1.1 mA activated 8.7% of the axons in the anisotropic model and 2.7 mA was required to activate 8.7% of the axons in the isotropic model. There were equivalent results for the SCP, where the anisotropic model predicted 26% of SCP axons activated at 1.2 mA through contact 4, while the isotropic model predicted 26% of SCP axons activated at 2.5 mA.

### Comparison of model simulations to PET imaging

**Figure 6:**
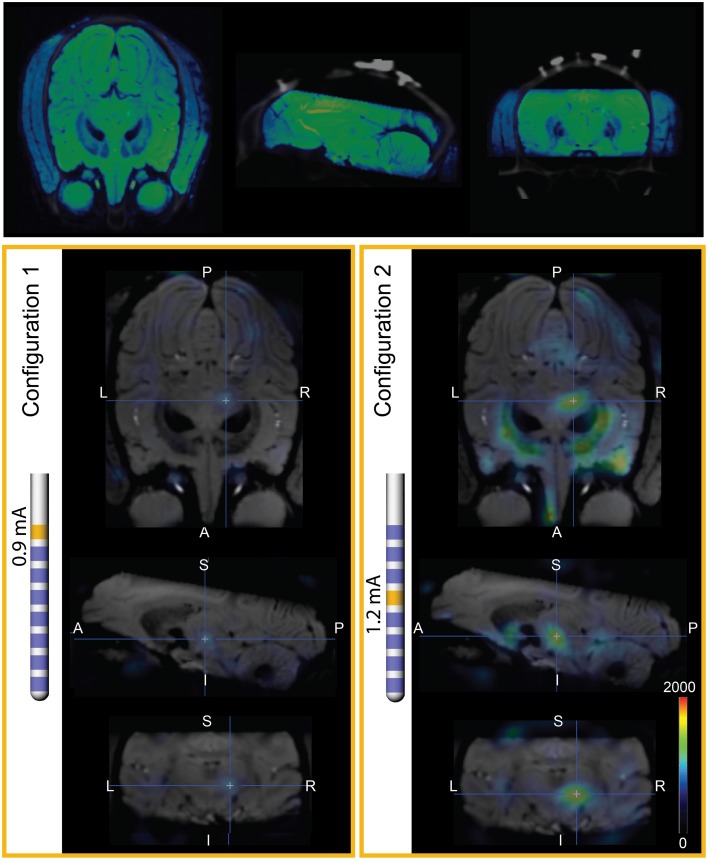
**PET imaging during PPTg-DBS. (Top)** Co-registration of SWI and baseline PET/CT images. The SWI is shown in blue-green cold scale for differentiation from the gray CT. **(Left)** FDG-SUV during PET configuration 1 (0.9 mA stimulation through contact 7), normalized to OFF-DBS. The PET results are overlaid on SWI of Monkey L. **(Right)** FDG-SUV during PET configuration 2 (1.2 mA stimulation through contact 4), normalized to OFF-DBS.

Two FDG-PET scans, in the context of DBS, were conducted to examine the effects of DBS in the PPTg area, after 0.9 mA stimulation through Contact 7 (configuration 1) and 1.2 mA stimulation through Contact 4 (configuration 2) (Tables 4, 5). These were compared to a baseline scan with no stimulation. A sampling of the resultant FDG standard uptake values (FDG-SUV) are shown in Figure 6. For the first stimulation configuration, the ventral posteromedial nucleus of thalamus (VPM), which is innervated by CTG (Blumenfeld, 2002), showed an increased FDG-SUV (*p* = 0.023). Further, descending projections of CTG project to the inferior olivary nuclei (Blumenfeld, 2002), which also showed an increased FDG-SUV in configuration 1 (*p* = 0.033). This corresponded to an activation of 12.2% of CTG fibers (Table 3). For configuration 2, the models predicted no activation of CTG; the PET measured a significant increase in FDG-SUV in the VPM (*p* = 0.041), but no significant increase in FDG-SUV in the inferior olivary complex (*p* > 0.05).

**Table 4:** **PET Configuration 1**.

**Region**	***P*-Value**	***T*-Score**
**CEREBELLUM**		
(R) Gracile lobule	0.044152	14.39561
(R) Simple lobule	0.030392	20.93132
(R) Inferior semilunar lobule	0.036188	17.57284
**THALAMUS**		
(R) Centromedian nucleus	0.036749	17.30426
(R) Ventral Posteromedial Nucleus (VPM)	0.022568	28.1971
**BRAINSTEM NUCLEI**		
(R) Interstitial nucleus of the vestibular nerve	0.019187	33.16989
(R) Deep mesencephalic nucleus	0.031026	20.50234
(R) Inferior olivary complex	0.032988	19.28151
(R) Lateral vestibular nucleus	0.041787	15.21301
(R) Abducens nucleus	0.03823	16.63219
(R) Spinal trigeminal nucleus—caudal part	0.04019	15.81927
(R) Nucleus of the bulbar accessory nerve	0.041015	15.50001
**GYRI**		
(R) Lingual gyrus	0.020319	31.32051
(L) Lingual gyrus	0.047783	13.29825
(L) Inferior occipital gyrus	0.035827	17.75039

**Table 5:** **PET Configuration 2**.

**Region**	**P-Value**	**T-Score**
**CEREBELLUM**		
(R) Lobule III	0.021944	28.99922
(L) Lobule III	0.027236	23.36013
(R) Flocculus	0.04999	12.70883
**THALAMUS**		
(R) Metathalamus	0.017863	35.62996
(R) Posterior intralaminar group	0.020822	30.56362
(L) Oral pulvinar nucleus	0.025265	25.18435
(R) Inferior pulvinar nucleus	0.029854	21.30901
(L) Ventral posteromedial nucleus—parvicellular part	0.04117	15.44166
**BRAINSTEM NUCLEI**		
(R) Inferior colliculus	0.015676	40.60312
(L) Gigantocellular nuclei	0.027296	23.30846
(L) Rostral interstitial nucleus of the medial longitudinal fasciculus	0.029331	21.68953
(R) Peripeduncular nucleus	0.034083	18.6606
(R) Rostral interstitial nucleus of the medial longitudinal fasciciulus	0.037915	16.77087
(L) Trochlear nucleus	0.040337	15.76126
(R) Red nucleus—Parvocellular part	0.040367	15.74982
(L) Brachium of the superior colliculus	0.0446	14.25069
(L) Central gray of the midbrain	0.045313	14.02564
(L) Reticulotegmental nucleus	0.046358	13.7083
(L) Interstitial nucleus of cajal	0.048904	12.99217
(L) Red nucleus—magnocellular part	0.048722	13.04081
(R) Retrorubral field	0.049879	12.73705
**BASAL GANGLIA**		
(R) Globus pallidus, internal (GPi)	0.016212	39.26104
(R) Globus pallidus, external (GPe)	0.025203	25.24599
(R) Nucleus accumbens	0.027752	22.92526
(L) Nucleus accumbens	0.033671	18.88927
(L) Subthalamic nucleus	0.045838	13.86446
**GYRI**		
(L) Medial orbital gyrus	0.025311	25.13825
(R) Posterior parahippocampal gyrus	0.038926	16.3343
(L) Straight gyrus	0.043415	14.6409
(L) Isthmus of the cingulate gyrus	0.047395	13.40732
**OTHER**		
(R) Amygdala	0.018961	33.56495
(R) Basal forebrain nucleus	0.025798	24.66311
(L) Field H	0.029511	21.55704
(L) Field H2	0.041625	15.27233
(R) Prepiriform cortex	0.030997	20.52183
(R) Olfactory tubercle	0.033287	19.10783
(L) Olfactory tubercle	0.049761	12.76748
(R) Hippocampus	0.034665	18.34666
(R) Substantia innominata	0.03942	16.12897
(R) Presubiculum	0.042929	14.80716
(L) Olfactory bulb	0.044335	14.33593
(R) Claustrum	0.047654	13.33429
(R) Mammillotegmental fasciculus	0.049034	12.99217

Similarly, regions that are innervated by projections from the PPTg showed an increased FDG-SUV, including the centromedian nucleus of thalamus (*p* = 0.037) during configuration 1. Configuration 2 showed increased FDG-SUV in regions innervated by fiber pathways near PPTg, including the rostral interstitial nucleus of the MLF and the interstitial nucleus of Cajal, which are innervated by MLF. Increased FDG-SUV in downstream targets of the PPTg was also observed in configuration 2, including the globus pallidus, basal amygdala, peripeduncular nucleus, centromedian nucleus, and the STN. The PET results also showed an increase in FDG-SUV in the red nucleus for configuration 2. This was supported by the model predicted activation of the SCP tract for configuration 2, 25.10% (Table 6).

**Table 6:** **Model comparison to behavior**.

**PET DBS Settings**	**SCP % Activated**	**CTG % Activated**
C7 (0.9 mA)	0	12.2
	–	–
	–	–
C4 (1.2 mA)	25.10	0

## Discussion

Subject-specific computational models are an important tool to better understand the mechanisms of DBS in the brainstem and guide future DBS therapies (Butson et al., 2007; Miocinovic et al., 2009; Chaturvedi et al., 2010; Keane et al., 2012; Lujan et al., 2012; Zitella et al., 2013). In order for these models to be clinically relevant they must provide accurate predictions. While other methods of validation have been applied to computational models of DBS, no models of the brainstem have yet been rendered subject specific. In this study, we evaluated the sensitivity of a subject-specific model of PPTg-DBS in a nonhuman primate to different model parameters (tissue conductance anisotropy, axonal diameter, and DBS lead location) and compared the results to behavioral and functional imaging measures to determine the most accurate tissue conductance model.

Our previous computational models assumed the DBS lead was surrounded by homogeneous, isotropic tissue with a conductivity of 0.3 S/m (Zitella et al., 2013). Based on the fractional anisotropy results from Monkey L and Monkey P in the brainstem, the mean of the image-based conductivity distribution did deviate from this isotropic conductivity assumption, but was well within an order of magnitude. Since the conductivity scaling factor did not greatly affect the model predictions, the spatial variability of the conductivity (i.e., the distribution of conductivities within the brainstem) proved to have a large effect on the potential distribution around the DBS lead. This high anisotropy near the lead resulted in lower stimulation amplitudes required to activate nearby axons despite the slightly higher average conductivity in the brainstem. Based on these results, it seems that anisotropy, in conjunction with the average conductivity, plays a role in the ability to activate axons.

Several other computational models of DBS have incorporated anisotropy of tissue conductivity, including models of STN DBS, which assigned typical conductivity values based on the literature (Sotiropoulos and Steinmetz, 2007; Åström et al., 2009). Other studies have converted fractional anisotropy measures from subject DTI to conductivity, including models of DBS in the STN region and in the thalamus (McIntyre et al., 2004a; Miocinovic et al., 2009). Similar to our models, regions with high anisotropy showed greater variability in the voltage isosurfaces and in the activated volume of tissue. However, these studies showed, contrary to our results, that the addition of anisotropy to the model decreased the percentage of axons activated. This difference may relate to axonal fiber orientations relative to the stimulated electrode(s) as well to assumptions of the neuron model parameters.

The PPTg area, similar to other typical DBS target regions, is highly anisotropic. Indeed, the PPTg is surrounded by the spinothalamic tract, CTG, medial lemniscus, lateral lemniscus, and the MLF. The fibers of the SCP are intertwined with the cells of the PPTg, which introduces challenges when attempting to stimulate one pathway over another. The present study showed that the inclusion of anisotropic conductivity is highly important for computational model predictions. This finding suggests that efforts to increase the resolution of fractional anisotropy imaging within the brainstem—through high-field, high angular resolution diffusion imaging (Lenglet et al., 2012), customized head coils (Adriany et al., 2010), and advanced computational reconstruction algorithms (Duarte-Carvajalino et al., 2014) as used in this study—could have significant merit (Novak et al., 2001; Stieltjes et al., 2001; Soria et al., 2011; Aggarwal et al., 2013; Deistung et al., 2013; Gizewski et al., 2014).

The clinical value of computational models lies in their legitimacy, making validation extremely important. Previous studies have confirmed the validity of their model parameters based upon correlations between EMG thresholds and activation of the corticospinal tract of internal capsule (during STN-DBS) (Butson et al., 2006; Chaturvedi et al., 2010), between paresthesia thresholds and activation of the somatosensory representation of thalamus (during Vim-DBS) (Kuncel et al., 2008), and between conjugate eye deviation and oculomotor nerve activation (during STN-DBS) (Butson et al., 2006). Here we extend this approach to the brainstem in the context of DBS targeting the PPTg area. The results showed much better predictions of the activation of the oculomotor nerve axons at stimulation amplitudes necessary to induce eyelid flutter for the anisotropic models. Without being able to measure the magnitude of the eyelid twitch or obtain verbal feedback on the strength of the side effect, this is a positive result. Additional modifications to the model equations, more precise anatomical geometry, and higher resolution DTI could provide more accurate results. The assumption that the conductivity is linearly related to the diffusion tensor eigenvalues may not hold for high resolution (1 mm voxels) DTI within the brainstem and could also be a source of error in the models.

In this study, PET imaging was used as a gross measure of the activation in the area during stimulation to compare the effects of stimulation through different contacts to baseline. PET is a valuable tool that has been used to examine the effects of DBS (Haslinger et al., 2003; Mayberg et al., 2005). The use of PET in the context of PPTg-DBS provided a novel approach to further evaluate the predictive capabilities of the computational neuron models. While the results were consistent, there are several limitations that should be noted. In addition to having only one subject, there was only one scan taken of each configuration (OFF DBS, C4 stimulation, C7 stimulation). Additional small spatial errors could also have been introduced when aligning the INIA19 atlas to the PET/CT. Furthermore, the PET analysis reported here did not account for the precise time from injection to time of scan. This will be incorporated in future studies for more accurate results.

Future studies will also need a larger sample size and expand the model validation methods. Through studies in nonhuman primates, the addition of electrophysiology would provide more insight into the effects of stimulation. The electrophysiological activation thresholds could be compared to the model predictions by recording single-unit spike activity at multiple sites within upstream and downstream targets of fiber pathways coursing near the PPTg.

As DBS techniques continue to advance, new targets are being explored and new lead designs are being developed. There is a growing need for validated computational models to better understand the therapeutic results and titrate stimulation parameters in human patients implanted with DBS systems. This study is the first case of incorporating anisotropic conductivity into subject-specific computational models of DBS in the brainstem. Moreover, the study emphasizes how coupling behavioral metrics and functional imaging data in computational modeling studies can be critical for enhancing the predictive power of the models.

## Conflict of interest statement

The authors declare that the research was conducted in the absence of any commercial or financial relationships that could be construed as a potential conflict of interest.
